# Laparoscopic Transplant Nephrectomy Using a Combination of Intracapsular and Extracapsular Approaches for Graft Intolerance Syndrome in Failed Renal Allografts

**DOI:** 10.1002/iju5.70237

**Published:** 2026-07-26

**Authors:** Yuto Naroda, Atsuhiko Ochi, Ryutaro Fukagai, Maiko Shimogawa, Masatoshi Matsunami, Jun Yashima, Tomo Suzuki, Yu Oyama, Hirokazu Abe, Masayoshi Okumi

**Affiliations:** ^1^ Department of Urology Kameda Medical Center Chiba Japan; ^2^ Renal Transplant Center Kameda Medical Center Chiba Japan; ^3^ Department of Nephrology Kameda Medical Center Chiba Japan; ^4^ Department of Nephrology Matsunami General Hospital Gifu Japan; ^5^ Department of Medical Oncology Kameda Medical Center Chiba Japan; ^6^ Department of Urology Kyoto Prefectural University of Medicine Kyoto Japan

**Keywords:** failed renal allograft, graft intolerance syndrome, kidney transplantation, laparoscopic surgery, transplant nephrectomy

## Abstract

**Introduction:**

Transplant nephrectomy for graft intolerance syndrome is technically challenging because severe perirenal adhesions increase bleeding risk. Despite the increasing adoption of minimally invasive techniques, optimal laparoscopic strategies remain limited.

**Case Presentation:**

A 49‐year‐old man developed pembrolizumab‐induced graft intolerance syndrome (GIS) following surgery for native renal cell carcinoma. To enable safe continuation of immunotherapy, laparoscopic transplant nephrectomy was performed. Despite severe adhesions, a combined intracapsular and extracapsular approach enabled safe graft removal. Multiple feeding vessels were divided using a vessel‐sealing device and soft coagulation, while avoiding extensive hilar dissection. Postoperative computed tomography confirmed complete resection of the allograph, with no radiologically evident residual tissue or postoperative complications.

**Conclusion:**

This hybrid laparoscopic approach is a safe, minimally invasive option for managing GIS with severe adhesions.

## Keynote Message

Transplant nephrectomy for graft intolerance syndrome is technically demanding because of severe perirenal adhesions. A combined intracapsular and extracapsular laparoscopic approach safely minimizes hazardous vascular dissection, achieves complete resection without radiologically evident residual allograft tissue, and facilitates the continuation of systemic therapy.

**Abbreviations**
CT: computed tomography
GIS: graft intolerance syndrome
ICI: immune checkpoint inhibitor
TN: transplant nephrectomy

## 1 Introduction

Transplant nephrectomy (TN) is indicated for various graft complications, including rejection, infection, vascular issues, or malignancies [1, 2]. Graft intolerance syndrome (GIS), characterized by pain, fever, and inflammatory symptoms, is the most common indication [1, 2]. Due to persistent perirenal inflammatory changes, TN for GIS is often accompanied by dense adhesions, increasing the risk of intraoperative bleeding and prolonged operative time [3, 4].

Open surgery (intracapsular or extracapsular) remains the standard approach [5]. Although minimally invasive approaches, including laparoscopic and robot‐assisted techniques, are increasingly applied [6, 7, 8], the optimal laparoscopic strategy for TN has not been fully established. Herein, we report a case of pembrolizumab‐induced GIS safely managed with laparoscopic TN using a combined intracapsular and extracapsular dissection approach, demonstrating a feasible strategy for managing complex adhesions.

## 2 Case Presentation

A 49‐year‐old man with graft failure at age 41 underwent staged laparoscopic bilateral radical nephrectomy for native renal cell carcinomas (pT3a and pT1a). Adjuvant pembrolizumab was initiated for the locally advanced left renal carcinoma. Six days later, the patient developed GIS, manifesting as high fever, chills, and graft‐site pain. Symptoms improved with steroid pulse therapy, but computed tomography (CT) showed enlargement of the previously atrophic graft (Figure 1). Laparoscopic TN was planned to enable safe continuation of immunotherapy. The temporal association strongly suggested that pembrolizumab‐induced immune activation triggered GIS.

**FIGURE 1:**
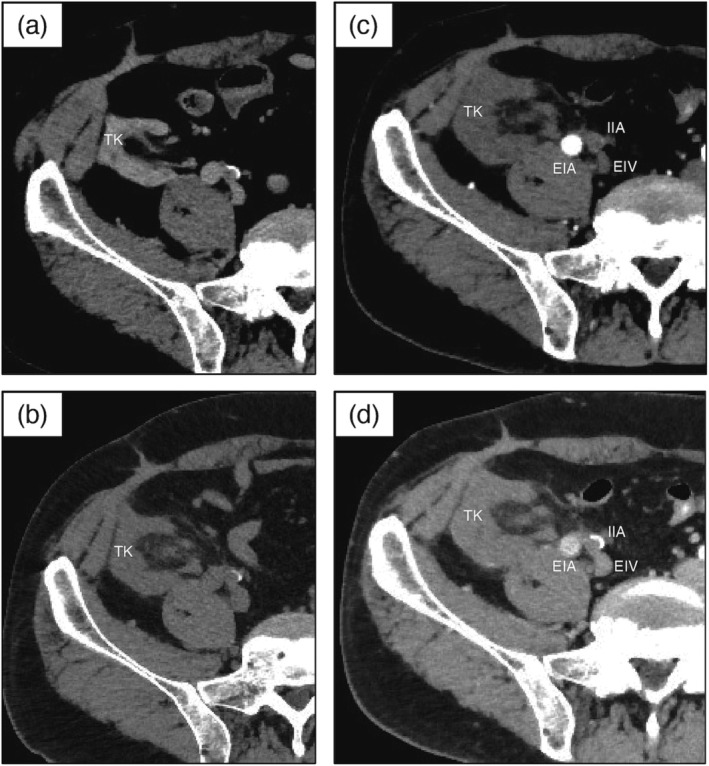
Computed tomography imaging of the failed renal allograft. (a) Atrophic failed renal allograft before immune checkpoint inhibitor therapy. (b) Enlargement of the failed renal allograft with increased perirenal fat stranding, consistent with graft intolerance syndrome, following the first dose of pembrolizumab. (c) Arterial‐phase imaging after steroid therapy showing persistent graft enlargement and markedly reduced flow in the internal iliac artery at the anastomotic site. (d) Equilibrium‐phase imaging after steroid therapy showing faint residual enhancement of the failed renal allograft. EIA, external iliac artery; EIV, external iliac vein; IIA, internal iliac artery; TK, transplanted kidney.

TN was performed laparoscopically using four ports in the left lateral position (Figure 2a). Intraperitoneal ultrasonography identified the vascular anastomosis, but severe adhesions limited further hilar dissection (Figure 2b–d). The peritoneum was incised along the clearly visible graft outline. Due to dense adhesions between the capsule and abdominal wall muscles, an extracapsular approach was employed to dissect circumferentially along the abdominal wall, delineating the hilum (Figure 3a–e). Subsequently, intracapsular dissection of the renal hilum was performed. Intraoperative exploration revealed multiple feeding vessels supplying the allograft, all of which were successfully divided using a vessel‐sealing device. The graft ureter was clipped and transected at the lower pole, and the kidney was removed (Figure 3f–j). Additional hemostasis was achieved using soft coagulation, and external iliac flow preservation was confirmed via ultrasonography (Figure 3k–o). The operative time was 3 h 15 min, with an estimated blood loss of 100 mL; no blood transfusion was required. Pathology revealed T‐cell‐mediated rejection.

**FIGURE 2:**
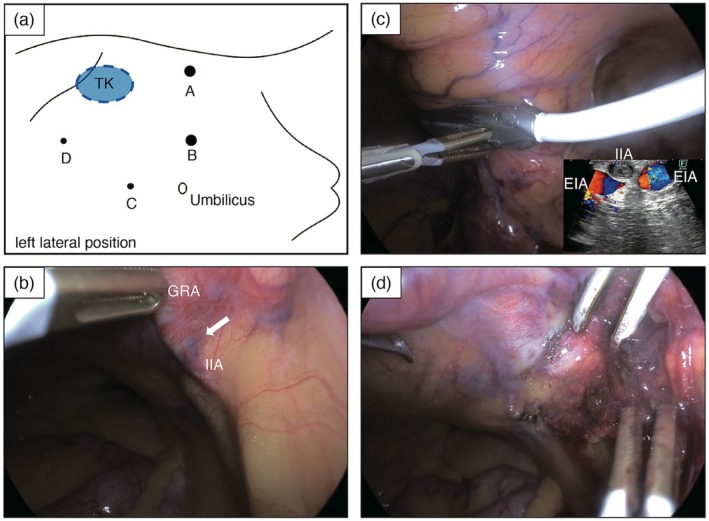
Port placement and intraoperative findings during laparoscopic transplant nephrectomy. (a) Port placement for laparoscopic transplant nephrectomy: (A) 12‐mm port (surgeon's right hand); (B) camera port; (C) 5‐mm port (surgeon's left hand); (D) 5‐mm port (assistant). (b) Laparoscopic view of the anastomosis between the graft renal artery and internal iliac artery, with vascular suture material identified. (c) Intraperitoneal ultrasonography showing the external iliac artery crossing posterior to the internal iliac artery, with markedly reduced flow in the internal iliac artery anastomosed to the graft renal artery. (d) Dense adhesions at the arterial anastomosis, making vascular dissection technically challenging. EIA, external iliac artery; GRA, graft renal artery; IIA, internal iliac artery; TK, transplanted kidney.

**FIGURE 3:**
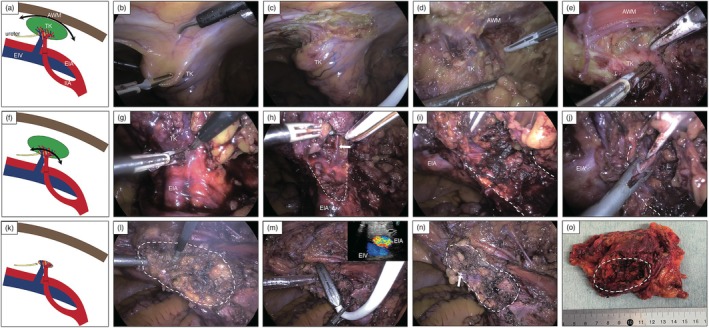
Surgical technique combining extracapsular and intracapsular approaches for laparoscopic transplant nephrectomy. (a) Schematic illustration of the extracapsular approach showing dissection between the renal capsule and abdominal wall muscles. (b) Outer margin of the failed renal allograft at the interface between the transplanted kidney and abdominal wall muscles before peritoneal incision. (c) The same region after peritoneal incision. (d) Dense adhesions between the graft renal capsule and abdominal wall fascia, resulting in capsular tearing during attempted dissection. (e) Dissection plane established along the graft renal capsule while preserving abdominal wall fascia, with exposure of muscle fibers. (f) Schematic illustration of the intracapsular approach showing dissection within the renal capsule at the hilum. (g) Incision of the renal capsule at the hilum adjacent to the external iliac artery. (h) Intracapsular dissection with division of small vessels (arrow) using a vessel‐sealing device. (i) Dissection proceeding from the upper pole to the lower pole of the transplanted kidney. (j) Clipping of the graft ureter prior to further dissection. (k) Schematic illustration after transplant nephrectomy showing removal of the renal allograft with preservation of the ureter and vascular structures. (l) Soft coagulation for hemostasis at the intracapsular resection surface of the renal hilum. (m) Intraperitoneal ultrasonography confirming preserved flow in the external iliac artery and vein. (n) Laparoscopic view at completion of the procedure (arrow indicates clipped ureter). (o) Resected transplanted kidney. Dashed line indicates the incised renal capsule edges at the hilum. AWM, abdominal wall muscles; EIA, external iliac artery; EIV, external iliac vein; IIA, internal iliac artery; TK, transplanted kidney.

Steroid therapy was discontinued, and the patient was discharged on day 5. Pembrolizumab was resumed on day 18. Follow‐up CT at 3 months confirmed no radiologically evident residual allograft tissue and preserved external iliac flow (Figure 4).

**FIGURE 4:**
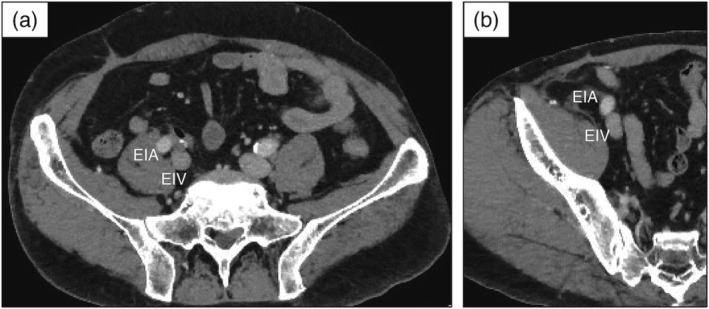
Computed tomography imaging after laparoscopic transplant nephrectomy. (a) Complete removal of the renal allograft. (b) Patent external iliac artery and vein without stenosis or thrombosis. EIA, external iliac artery; EIV, external iliac vein.

## 3 Discussion

TN for GIS is technically challenging because chronic inflammation results in dense perirenal adhesions, increasing bleeding risks. Major complications occur in up to 21% of late TNs [1], reflecting high morbidity. Therefore, the choice of dissection strategy is critical. In this case, laparoscopic TN was performed using a combined approach for GIS following immunotherapy, demonstrating the safety of this strategy.

The intracapsular approach is often selected for late cases with severe adhesions, whereas the extracapsular approach is preferred in early cases [1, 2, 4]. Here, direct hilar dissection was unsafe due to dense adhesions. Therefore, we initiated an extracapsular approach from the abdominal wall, establishing a safe dissection plane that avoided capsular tearing and reduced bleeding. After outlining the graft, selective intracapsular resection was performed at the hilum, avoiding direct dissection of densely adherent major vessels and reducing vascular injury risks.

Regarding the vascular dissection strategy in the robotic approach, identifying the common iliac artery first and subsequently proceeding toward the vascular anastomosis has been recommended [7]. Although this strategy facilitates proximal vascular control, we intentionally limited the extent of dissection in this case. Dense perienteric adhesions from previous surgery and persistent inflammation made extensive retroperitoneal vascular dissection likely to increase the risk of injury to adjacent vessels and organs, including the small intestine and colon. Therefore, we minimized the dissection area to prioritize procedural safety.

With respect to arterial stump management, conventional open intracapsular TN often requires vascular clips or suture ligation to prevent delayed hemorrhage or pseudoaneurysm formation. In our case, however, intraoperative exploration identified multiple feeding vessels to the allograft rather than a single dominant artery. These vessels were safely divided using a vessel‐sealing device, followed by soft coagulation to ensure hemostasis. This approach avoided the need for mechanical clips or sutures in a densely adherent and fragile operative field, and postoperative imaging demonstrated no vascular complications.

Despite severe adhesions, the operative time (195 min) and blood loss (100 mL) compare favorably with previous series. Embolization or open TN may be considered as alternatives; however, embolization may not provide definitive symptom control, while open surgery has higher morbidity.

Continuation of ICI therapy was essential in this case. Minimally invasive TN reduces surgical stress, facilitating early return to systemic therapy [6, 7, 8]. As ICI‐induced GIS can recur even in nonfunctioning allografts, definitive and minimally invasive removal is clinically valuable. This report is limited by its single‐case design.

In conclusion, laparoscopic TN using a combined extracapsular and intracapsular approach is a safe, effective option for managing GIS with severe adhesions. This strategy minimizes vascular dissection, achieves complete resection without radiologically evident residual allograft tissue, and facilitates rapid postoperative recovery.

## Ethics Statement

This case report was approved by the Board of Ethics of the Kameda Medical Center (25‐028).

## Consent

Informed consent for publication was obtained from the patient.

## Conflicts of Interest

The authors declare no conflicts of interest.

## Data Availability

The data that support the findings of this study are available from the corresponding author upon reasonable request.
